# A practical approach to mentoring students with repeated performance deficiencies

**DOI:** 10.1186/1472-6920-13-56

**Published:** 2013-04-19

**Authors:** Kevin McLaughlin, Pamela Veale, Joann McIlwrick, Janet de Groot, Bruce Wright

**Affiliations:** 1 Office of Undergraduate Medical Education, University of Calgary, Calgary, AB, Canada; 2 Office of Equity and Teacher-Learner Relations, University of Calgary, Calgary, AB, Canada

## Abstract

**Background:**

With the increasing use of competency-based evaluations we now have more and better ways to identify performance deficiencies in our learners. Yet the emphasis placed on identifying deficiencies appears to exceed that given to improving these deficiencies.

**Aims:**

Here we describe the program at the University of Calgary for mentoring students with repeated performance deficiencies. We focus primarily on the key steps of mentoring and remediation, and establishing a program that provides consistency and accountability to this process.

**Conclusions:**

A small cohort of trainees with persistent performance deficiencies may need intensive remediation to reach the expected level of performance. Ultimately, not all learners will be successful in their remediation, but we feel that it is the responsibility of training programs to provide mentorship and an organized approach to remediation in order to maximize the chances of successful remediation.

## 

With the increasing use of competency-based evaluations, including the objective structure clinical examination (OSCE) and the in-training evaluation report (ITER), we now have more and better ways to identify performance deficiencies in our learners [1]. Yet it appears that our proficiency in identifying deficiencies is not matched by readiness to remediate these [2-4]. Fortunately, most learners are at or above the minimum performance level (MPL), and those who dip below this level can usually raise their performance on their own. But a small number of our learners are unable to self-remediate and have persistent performance deficiencies that prevent them from advancing through their training. So, are we, as medical educators, deficient in dealing with these learners? And, if so, how can we improve our performance?

At the University of Calgary we have developed a formal mentoring program for students with repeated (i.e., ≥ 2) “unsatisfactory” or “performance deficiency” ratings on undergraduate courses or clerkship rotations, and the purpose of this article is to describe the philosophy of our program and to provide practical advice for other schools considering such a program. Although our program deals with undergraduate students these suggestions are also relevant to learners at more advanced stages of training.

## Only begin mentoring when barriers to successful remediation have been removed

Students with repeated academic and/or non-academic problems frequently have other problems that may impair their ability to successfully remediate, including physical or mental health problems, substance abuse, learning disabilities, fatigue, or financial concerns [5-7]. Therefore, before accepting a student into our remediation program we require a letter from the referring individual (either the associate dean or an assistant dean of undergraduate medical education) stating that the student does not have active problems that will interfere with their ability to remediate. If such a problem arises during the remediation period – for example if the student takes a medical leave of absence – the remediation process stops until the student is re-referred with a letter stating that this problem is resolved.

## Select a mentor who is unbiased

Failing students are often concerned that their reputation precedes them so that future teachers and/or evaluators will be unduly biased against them. While it may not be possible to guarantee a *tabula rasa* for all students, we strive to select mentors who are free of bias towards the student. As such, we exclude teachers who have been involved in previous unsatisfactory evaluations, are likely to be future evaluators, or are in a position of authority, such as clerkship director, evaluation coordinator, or associate dean. We create a list of potentially suitable mentors – i.e., those we feel have relevant content expertise and no apparent bias – and the student then ranks these individuals. We then invite potential mentors, beginning with the highest ranked individual.

## Clarify expectations of the mentor

In our program the role of the mentor is focused and clearly defined: to identify the problems that led to the student’s unsatisfactory evaluation(s), to propose a process of remediation, and to supervise this process. The mentor is not responsible for dealing with barriers to remediation (see Tip 1), and does not determine the conditions of remedial clinical rotations or evaluations. In our program we specifically avoid asking the mentor to take on the role of evaluator. Thus, although the mentoring role is formal, the mentor does not provide a formal evaluation of the student’s progress.

## Clarify expectations of the student

We also define the expectations of the remediating student: to disclose all relevant information on previous performance, to allow access to all prior evaluations, and, where indicated, to allow the mentor to gather further information – either by interviewing staff or by directly observing their performance. If the student agrees with the mentor on the reasons for their poor performance and the proposed plan for remediation, they are then expected to participate fully in remediation activities.

## Confirm the nature and cause of the performance deficiencies

Successful remediation begins with an accurate diagnosis of the nature and cause of the student’s performance deficiencies. Mentors begin this process by reviewing all of the student’s formal evaluations. In the preclinical years this is relatively straightforward as students have a limited number of evaluations and are referred primarily due to performance deficiencies on knowledge evaluations. Competency-based evaluations in clerkship, however, sample more than knowledge (or the Medical Expert Role) [8] – thus increasing the likelihood of students being referred for remediation of non-Medical Expert deficiencies, or a combination of deficiencies. Where the mentor feels that there are insufficient data to diagnose deficiencies in the Medical Expert Role she can directly observe the student’s performance with real or standardized patients, or via simulation. The mentor may also interview former preceptors/evaluators, particularly if deficiencies lie in roles other than the Medical Expert.

## Create a learning plan with the end in mind

Although we are strong advocates of life-long learning, the mentoring process is driven by a short-term goal: for the student to be successful on his remedial evaluations. Thus, the objectives for remediation must be congruent with these evaluations. To facilitate congruence – or *content validity* – between remediation and evaluation, we encourage both mentor and student to review the evaluation blueprint if the remedial evaluation is a multiple choice question examination or OSCE, or the ITER rating form that will be used to evaluate performance on a clinical rotation [9]. Thus, when creating the learning plan the mentor can focus on performance deficiencies that will be re-evaluated.

## Identify appropriate training activities

Having identified which aspects of performance are deficient and will be re-evaluated, the next task for the mentor is to identify appropriate training activities to improve these deficiencies. There are a wide variety of solutions available that should be tailored to the specific problems. Table 1 shows the typical training activities recommended for the various types of performance deficiencies.

**Table 1:** Typical training activities, assessment, and feedback for specific performance deficiencies

**Performance deficiency**	**Training activity**	**Assessment of progress**	**Mechanism of feedback**
***Knowledge***	Self-study with advice on resources & study technique	Knowledge evaluations	Evaluations reviewed with mentor and feedback provided
***History taking***	Real or standardized patients	Direct observation	Immediate feedback
***Physical examination***	Real, standardized, or simulated patients	Direct observation	Immediate feedback
***Clinical reasoning***	Paper or online cases	Direct observation of student “thinking aloud”	Immediate feedback
***Communication***	Real or standardized patients	Direct observation	Immediate feedback
***Collaboration***	Clinical rotations	Weekly evaluations from clinical preceptor	Evaluations reviewed with mentor and feedback provided
***Manager***	Clinical rotations	Weekly focused evaluations from clinical preceptor	Evaluations reviewed with mentor and feedback provided
***Health Advocate***	Clinical rotations	Weekly focused evaluations from clinical preceptor	Evaluations reviewed with mentor and feedback provided
***Scholar***	Clinical rotations	Weekly focused evaluations from clinical preceptor	Evaluations reviewed with mentor and feedback provided
***Professionalism***	Clinical rotations	Weekly focused evaluations from clinical preceptor	Evaluations reviewed with mentor and feedback provided

## Follow the principles of deliberate practice

Unsupervised practice, even with well-designed training activities, is unlikely to remediate persistent performance deficiencies. The goal of the mentoring process is to create the optimum training conditions that Ericcson refers to as “deliberate practice” [10,11]. Thus, the appropriate training activities should be supplemented by direct observation, immediate feedback, and the opportunity for further practice. In reality, however, where the deficiencies lie in roles other than the Medical Expert, and the appropriate training activity is to repeat a clinical rotation, mentors may not be able to provide direct observation and immediate feedback, instead relying on preceptors to perform this role. Table 1 shows how we typically try to assess the student’s progress and provide feedback for each type of performance deficiency.

## Establish a timeline with intermediate goals

As the goal of mentoring is for the student to pass his summative evaluation, the mentoring process usually has a defined timeline that ends with this evaluation. Knowing where the student’s performance begins, where this should be – and by when – allows the mentor to create a timeline with intermediate goals. These goals should be congruent with the summative evaluation and progressively increase until the MPL is reached or exceeded.

## Create a mentoring contract

Following the first nine tips prepares the mentor and student for mentoring – but success of this process requires a commitment from both to follow the learning plan. Our mentoring “contract” is in the form of a letter from the mentor to the referring assistant or associate dean outlining the perceived deficiencies, details or the learning plan – including training activities and frequency of meetings – and documenting that both mentor and student agree with this plan. A copy to this letter is also given to the student. And now the mentoring begins…

## Monitor progress and revise the learning plan

The axiom of Robert Burns that *“The best laid schemes o' mice an' men gang aft agley…”* applies to mentoring students with performance deficiencies, and mentors and students should be prepared to change the learning plan, as needed, through the remediation process. In anticipation of this, the mentor should document when meetings took place, which activities occurred, the student’s progress, and any revisions to the learning plan. In the event of the student being unsuccessful at the end of the remediation period this also serves as documentation of the efforts of the training program to tailor remedial activities to the specific learning needs of the student.

## Allow either mentor or student to end the mentoring relationship

Even with the best of intentions, planning, and effort, the mentor and student may not be compatible. In this case, either the mentor or the student may dissolve the mentoring relationship. To ensure that both mentor and student feel free to break their relationship, dissolution is always deemed “no fault”, and the student then meets with the director of the mentoring program to select an alternative mentor.

## Update on our program

We introduced our program in September 2011. Our mentors are drawn from a pool of Master Teachers who spend 10 – 20% of their time teaching in the undergraduate program. The Master Teachers have a variety of clinical backgrounds, and each potential mentors has received further training in education theory, teaching, and conflict resolution. Rather than having a fixed schedule of meeting between student and mentor, the required frequency of meetings is decided by the individual student and mentor. During each meeting with their mentor students are asked to provide an assessment of their progress. The students and their mentor then review the latest *third party* ratings (e.g., ITER, OSCE, or MCQ examination) of the student’s performance in an attempt to calibrate the student’s self-assessment and/or explain discrepancies between the student’s rating and third party rating. Recognizing the importance of social support and social learning, in addition to meeting with their mentor, students are also encouraged to participate in a “Study Buddy” program [12,13]. In this program students join one or more fellow students in a study group where they actively participate in teaching each other and in preparing for examinations [14].

Thus far five students have participated in our mentoring program. Two students were in clerkship and were felt to have significant and persistent deficiencies in knowledge and clinical reasoning. Both of these students were successful during their remedial rotations and went on to graduate and begin residency training. Three students were referred with repeated academic performance in their first year. Of these, one is currently in the second year and is still being mentored. The other two students have been promoted to clerkship without further academic difficulties. We have since revised our inclusion criteria for mentoring so that clerkship directors or assistant deans can refer a student for mentoring before they have demonstrated repeated performance deficiencies.

## Conclusion

According to Rita Mae Brown (and perhaps Benjamin Franklin and Albert Einstein before her) insanity is *“…doing the same thing over and over again and expecting different results.”* Thus, to best serve learners with persistent performance deficiencies we need to modify their training. Given the well-documented reluctance to fail learners [15], the small cohort of trainees with persistent performance deficiencies may have major problems, and need intensive remediation to reach the expected level of performance. Yet we feel that investing this effort is preferable to the Spartan approach of allowing learners to continue to fail and then withdraw from their training program. Ultimately, not all learners will be successful in their remediation, but providing mentorship and an organized approach to remediation can at least improve their chances.

## Competing interests

The authors declare that they have no competing interests.

## Pre-publication history

The pre-publication history for this paper can be accessed here:

http://www.biomedcentral.com/1472-6920/13/56/prepub
